# Screening for proteins related to the biosynthesis of hispidin and its derivatives in *Phellinus igniarius* using iTRAQ proteomic analysis

**DOI:** 10.1186/s12866-021-02134-0

**Published:** 2021-03-12

**Authors:** Jinjing Guo, Xiaoxi Liu, Yuanjie Li, Hongyan Ji, Cheng Liu, Li Zhou, Yu Huang, Changcai Bai, Zhibo Jiang, Xiuli Wu

**Affiliations:** 1 College of Pharmacy, Ningxia Medical University, Yinchuan, 750004 P.R. China; 2 Department of Pharmaceutics, General Hospital of Ningxia Medical University, Yinchuan, 750004 P.R. China; 3 Key Laboratory for Chemical Engineering and Technology, State Ethnic Affairs Commission, School of Chemistry and Chemical Engineering, North Minzu University, Yinchuan, 750021 P.R. China

**Keywords:** *Phellinus igniarius*, Biosynthesis, Hispidin, iTRAQ, Proteomic

## Abstract

**Background:**

Hispidin (HIP) and its derivatives, a class of natural fungal metabolites, possess complex chemical structures with extensive pharmacological activities. *Phellinus igniarius*, the most common source of HIP, can be used as both medicine and food. However, the biosynthetic pathway of HIP in *P. igniarius* remains unclear and we have a limited understanding of the regulatory mechanisms related to HIP. In this work, we sought to illustrate a biosynthesis system for hispidin and its derivatives at the protein level.

**Results:**

We found that tricetolatone (TL) is a key biosynthetic precursor in the biosynthetic pathway of hispidin and that its addition led to increased production of hispidin and various hispidin derivatives. Based on the changes in the concentrations of precursors and intermediates, key timepoints in the biosynthetic process were identified. We used isobaric tags for relative and absolute quantification (iTRAQ) to study dynamic changes of related proteins in vitro. The 270 differentially expressed proteins were determined by GO enrichment analysis to be primarily related to energy metabolism, oxidative phosphorylation, and environmental stress responses after TL supplementation. The differentially expressed proteins were related to ATP synthase, NAD binding protein, oxidoreductase, and other elements associated with electron transfer and dehydrogenation reactions during the biosynthesis of hispidin and its derivatives. Multiple reaction monitoring (MRM) technology was used to selectively verify the iTRAQ results, leading us to screen 11 proteins that were predicted to be related to the biosynthesis pathways.

**Conclution:**

These findings help to clarify the molecular mechanism of biosynthesis of hispidin and its derivatives and may serve as a foundation for future strategies to identify new hispidin derivatives.

**Supplementary Information:**

The online version contains supplementary material available at 10.1186/s12866-021-02134-0.

## Background

Edible and medicinal fungi are a common source of nutrients and medicines in Asian nations and are well recognized as an important source of biologically active compounds. *Phellinus igniarius* (DC.Ex Fr.) Quel, a wild macrofungi, contains many bioactive compounds with properties such as antibacterial, antioxidative, antitumor, and antimutagenic activities, and has been widely used in China, Japan, and Korea for many years [1–3]. *P. igniarius* is rich in secondary metabolites with biological activities, such as polysaccharides, polyphenols, flavonoids, and other chemical species [4, 5]. Phelligridins are a class of chemical compounds from *P. igniarius* that possess radical-scavenging activity and a broad range of pharmacological properties, and includes chemicals such as Phelligridin D and Phelligridin G [6, 7]. Based on structural analyses, it has been postulated that hispidin is a key intermediate of phelligridins [8]. Hispidin (6-(3,4-dihydroxystyryl)-4-hydroxy-2-pyrone, HIP), is an important polyphenol found in *P. igniarius*. Hispidin is widely distributed in edible mushrooms, with many reports of potential pharmacologic value in the literature [9, 10]. Prior reports have suggested that HIP has activities such as anti-oxidation [11], anti-inflammatory [12], anti-cancer [13], and antiallergic [14] effects, among others. HIP is also an important luciferin precursor in some bioluminescent fungi and can be used for bioluminescent analysis of substances [15, 16]. A dose of 3 mg/g hispidin-enriched fungus mycelia has a very low level of toxicity, supporting its safety for human consumption [17]. Therefore, HIP has attracted significant attention in the fields of chemistry, pharmacy, and microbiology [18–21].

**Fig. 1:**
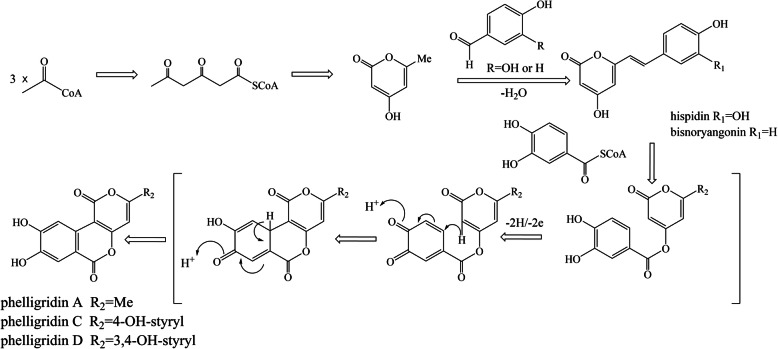
The proposed biosynthesis of HIPs

Due to the limited and unpredictable supply of wild *P. igniarius*, artificial fermentation has become a topic of increasing interest in recent years. Significant research has focused on optimizing fermentation conditions with the goal of increasing the yield of HIP [17]. With the development of sequencing technology, gene expression control at the transcriptional level is one method to determine composition and facilitate industrial production. Knowledge of the biosynthetic pathways of metabolites provides an important theoretical basis for such engineering approaches. To date, although the biosynthetic pathway of HIP and its derivatives in *P. igniarius* has been the focus of extensive research, the exact mechanisms and pathways remain incompletely understood. A previous study proposed the biosynthesis of HIP as shown in Fig. 1, where HIP is formed by the condensation of 4-hydroxy-6-methyl-2-pyrone (TL) and one molecule of 3,4-dihydroxybenzoyl-SCoA (or 3,4-dihydroxybenzaldehyde) in the *P. igniarius* fruit body [22]. This hypothesis has not been verified, and the biosynthetic pathway of HIP has not been categorically confirmed.

This work investigated whether TL is transformed into HIP in the fermentation broth of P. igniarius. Moreover, the extent of protein expression by the mycelium of *P. igniarius* was measured using iTRAQ technology following TL feeding. Proteins related to HIP synthesis were identified. The analysis explored how each protein participated following TL precursor feeding, and provides deeper insights into the mechanisms of synthesis of HIP at the proteomic level.

## Results

### Metabolic analysis method determination

**Fig. 2:**
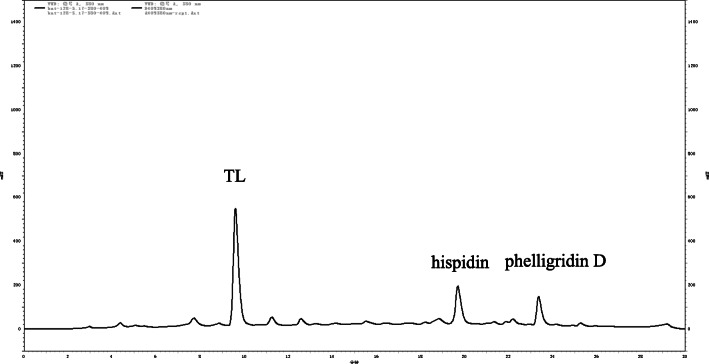
The metabolites after cultured for 7 days after TL feeding

An HPLC method was optimized to distinguish between HIP and other derivatives in order to analyze the ethyl acetate extracts. As seen in Fig. 2, the peak at 19.7 min was attributed to HIP, 23.4 min was phelligridin D, and 9.6 min was TL. To determine that the peaks at 19.7 min and 23.4 min were indeed HIP and phelligridin D, the chemical structures of the two purified compounds **A** and **B** were assessed by NMR spectra and HRESIMS (see Fig. S1–9).

Compound **A** was obtained as a light-yellow powder. Its quasi-molecular ion peak, observed at *m/z* 245.0453 (cal. 245.0450) [M - H]^−^ by HRESIMS and NMR data, showed the molecular formula as C_13_H_10_O_5_. The ^1^H-NMR spectrum of **A** in CH_3_OD (see Fig. S3) displayed resonances in pairs attributable to two analogous compounds with a content ratio of 3:2. This difference was determined to result from the *cis* and *trans* double bonds and some changes in chemical shift. The resonances were ascribed to two ABX spin coupling systems of 1,3,4-trisubstituted phenyl moiety at *δ*_H_ 6.95 (dd, *J* = 2.0, 8.0 Hz), 6.77 (d, *J* = 8.0 Hz), 7.03 (d, *J* = 2.0 Hz), and *δ*_H_ 6.80 (dd, *J* = 2.0, 8.0 Hz), 6.73 (d, *J* = 8.0 Hz), 6.98 (d, *J* = 2.0 Hz), a *trans*-disubstituted and a *cis*-disubstituted double bond at *δ*_H_ 7.31 (d, *J* = 16.0 Hz), 6.60 (d, *J* = 16.0 Hz), and 6.72 (d, *J* = 12.8 Hz), 5.98 (d, *J* = 12.8 Hz). The ^13^C-NMR spectrum of **A** gave two sets of 12 sp^2^ carbon resonances. Compared with the peak position of standards in HPLC and the data from HRESIMS, the compound was speculated to be a combination of *trans*-hispidin and *cis*-hispidin (see Fig. S1), which was also confirmed by 2D NMR. Analysis of gHSQC spectroscopic data of A furnished assignments of the proton-bearing carbon and corresponding proton resonances in the NMR spectra (Table 1). Although the signals of H_3_ and C_3_ disappeared in NMR, the visible long-range heteronuclear correlations in the HMBC of *trans*-hispidin for H-5/C-3 (~ 90 ppm, see Fig. S7) and the data in the literature [11, 23] supported the prior findings. Regardless of whether it is *trans* or *cis*, hispidin is a crucial intermediate of phelligridins [22].

**Table 1:** NMR spectroscopic data of compound **A**^*a*)^

position	***trans-***		***cis***-	
	*δ*_H_	*δ*_C_	*δ*_H_	*δ*_C_
2		173.9		173.5
3	*	90.0^*b*)^	*	*
4		168.0		168.1
5	6.11 s	102.1	6.13 s	104.0
6		162.2		162.1
7	6.60 d *J* = 16.0 Hz	117.1	5.98 d *J* = 12.8 Hz	119.0
8	7.31 d *J* = 16.0 Hz	137.4	6.72 d *J* = 12.8 Hz	139.2
9		129.0		128.8
10	7.03 d *J* = 2.0 Hz	115.0	6.98 d *J* = 2.0 Hz	117.2
11		147.0		146.3
12		148.8		147.8
13	6.77 d *J* = 8.0 Hz	116.7	6.73 d *J* = 8.0 Hz	116.3
14	6.95 dd *J* = 8.0, 2.0 Hz	122.1	6.80 dd *J* = 8.0, 2.0 Hz	123.4

*a*) Data (*δ*) were measured in CH_3_OD at 400 MHz for protons and at 100 MHz for carbons. Proton coupling constants (*J*) in Hz are given in parentheses. The assignments were based on ^1^H-^1^H COSY, HSQC, and HMBC experiments.* Indicates the signal is not detected. *b*) Signals were extracted from HMBC spectrum

Compound **B** was obtained as a yellow powder. Its quasi-molecular ion peak, found at *m/z* 379.0466 (cal. 379.0454) [M - H]^−^ by HRESIMS, gave a molecular formula of C_20_H_12_O_8_. Compound **B** was identified as phelligridin D by comparing with standards and data from ^1^H-NMR spectra (see Fig. S9) [22].

### The dynamic changes in concentration of TL and HIP

**Fig. 3:**
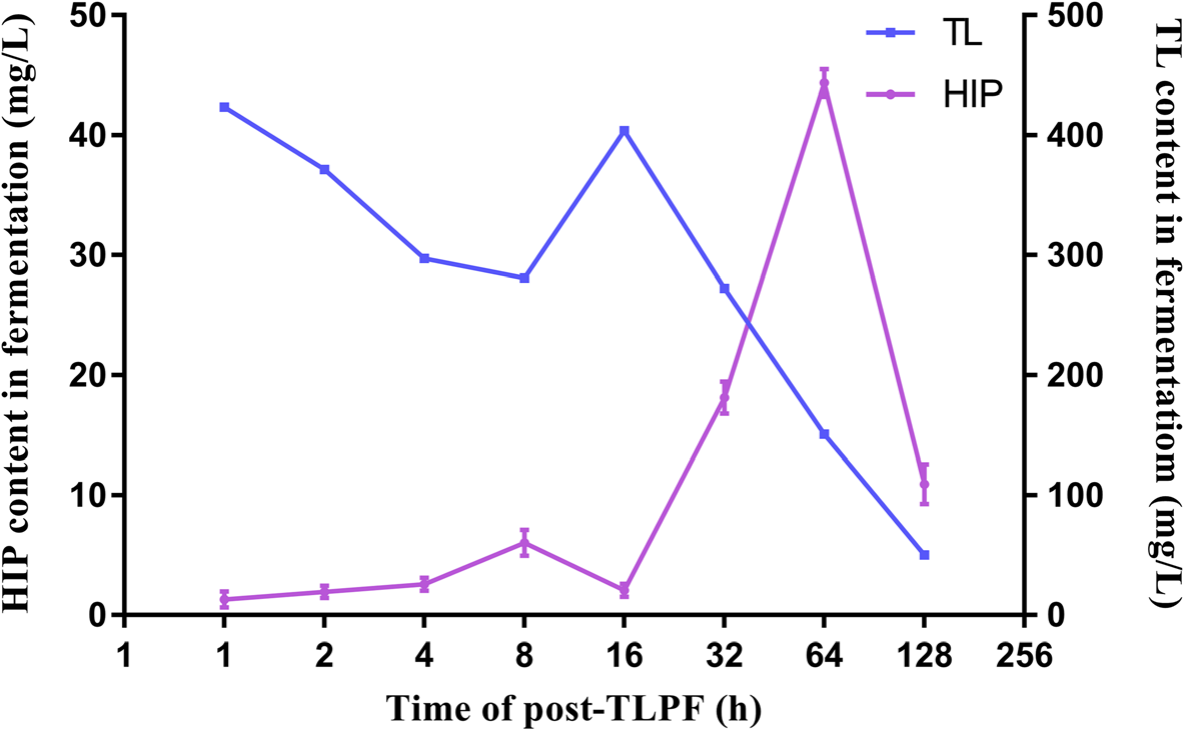
The dynamic changes in concentration of TL and HIP

Compared to the negative control condition that was not given TL, the contents of HIP were increased five-fold three days after the addition of TL, and the TL content gradually decreased (Fig. 3), suggesting that TL participates in the biosynthesis of HIP. Interesting, the content of TL and hispidin, from 8 to 16 h after TL feeding, ran contrary to the general trends observed over the longer time period, suggesting that the strain was in the logarithmic phase of growth through this period. Hispidin content rose sharply over the course of 16–64 h, from < 5 mg/L at 16 h to 44.38 mg/L at 64 h. Interestingly, TL rapidly decreased during the first 128 h after addition, wherein the concentration of TL declined from 423.61 mg/L at 1 h to 50.44 mg/L at 128 h), suggesting that TL may be consumed to synthesize hispidin. After 64 h, the concentration of HIP began to decline, presumably due to the synthesis of other derivatives.

### iTRAQ protein analysis

**Fig. 4:**
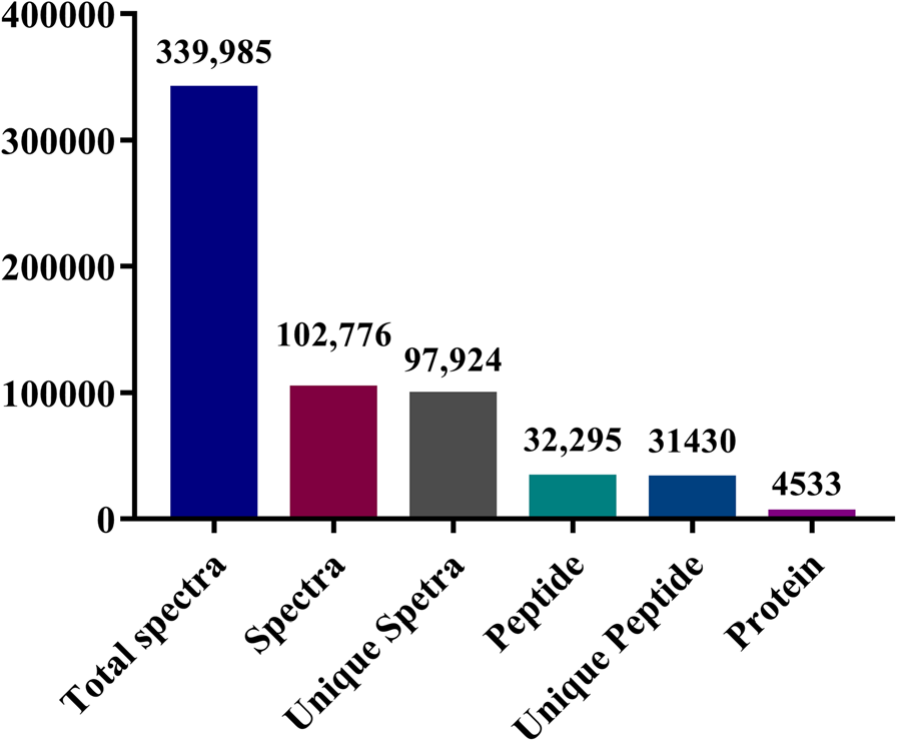
Overview of protein identification

**Fig. 5:**
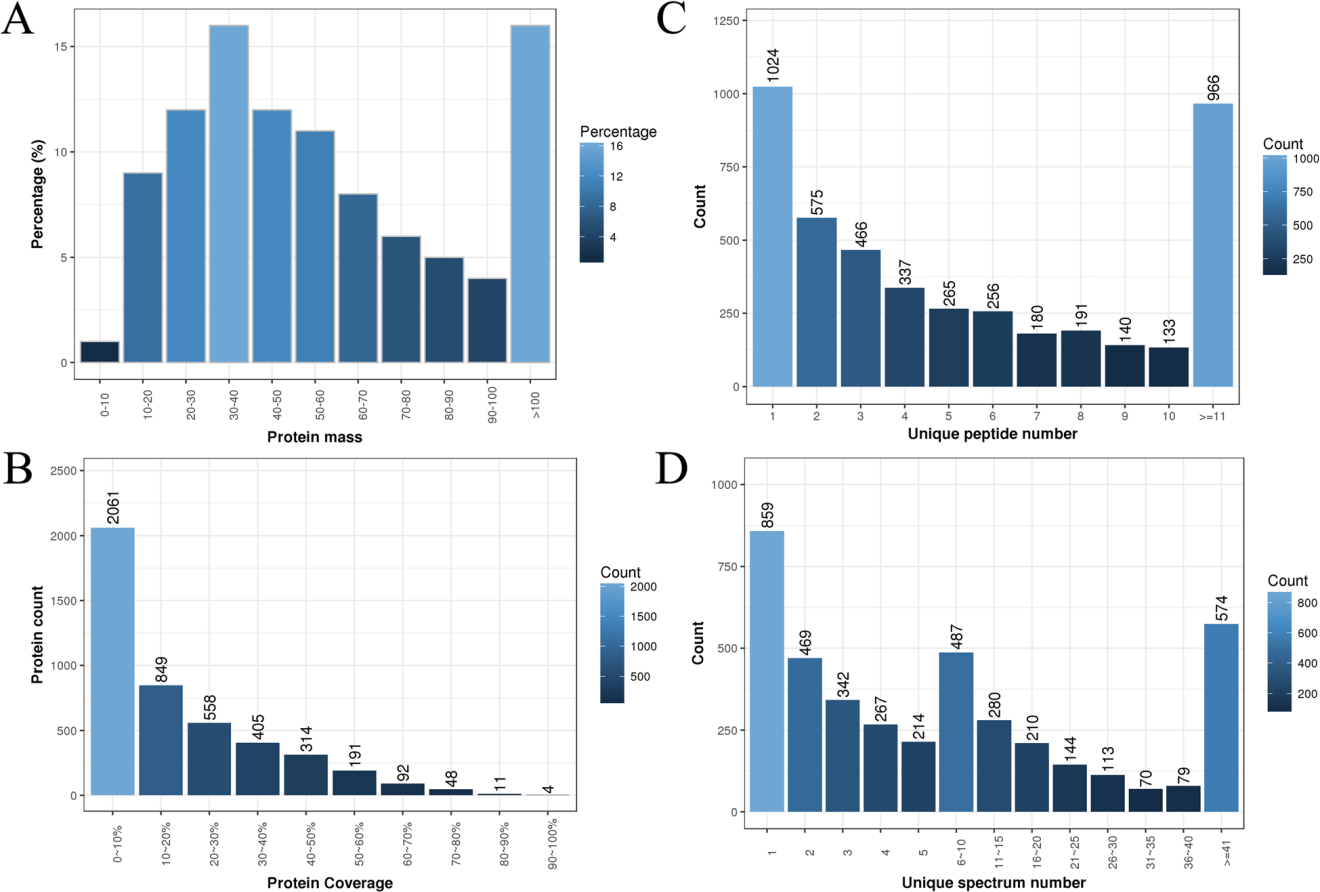
Information for protein identification. **a**: protein quantity distribution; **b**: identified protein coverage; **c**: number of specific peptide segments; **d**: specific peptide number

Herein, the expression profiles of proteins that were produced by *P. igniarius* that was fed with either TL-supplemented or normal media were compared by iTRAQ analysis. A total of 339,985 spectrums were filtered by 1% FDR, which led to the identification of 5630 peptides and 1880 proteins (Fig. 4). These results indicated that iTRAQ had higher sensitivity and provided more comprehensive information than other techniques in the analysis of these fungal proteins. The molecular weights of the identified proteins were widely distributed, mostly falling between 10 and 70 kDa. There were 552 proteins with molecular weights between 20 and 30 kDa, 794 proteins between 30 and 40 kDa, 543 between 40 and 50 kDa, and 710 over 100 kDa (Fig. 5a and b). Protein coverage analysis showed that the numerical value from 0 to 100% gradually declined. There were 966 proteins with more than 11 specific peptide segments. The number of specific spectra is shown in Fig. 5c. The number distribution of specific peptide segments is shown in Fig. 5d, and suggests that the results from this analysis were generally reliable.

### Analysis of the difference expression of proteins (DEPs)

**Fig. 6:**
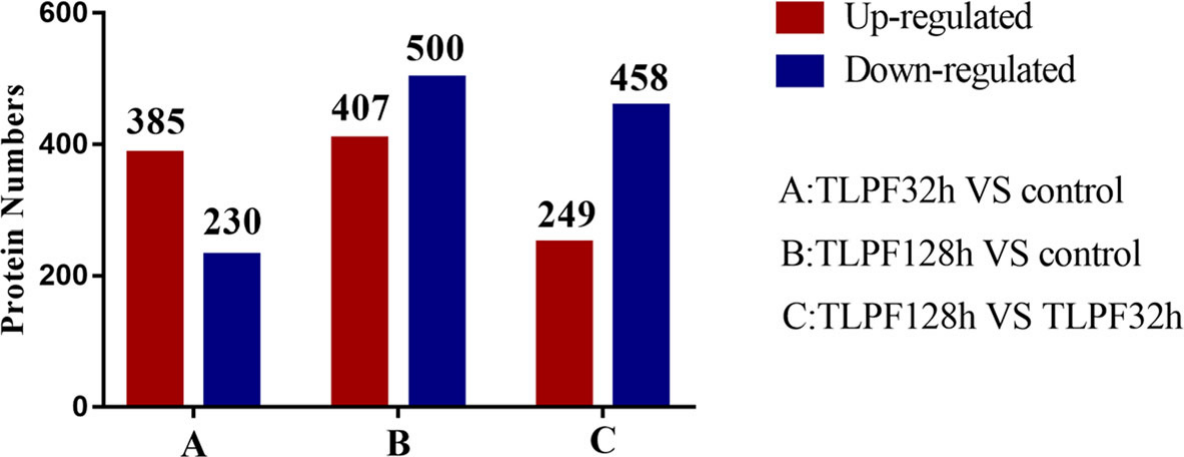
Three sets of differentially expressed proteins (DEPs). **a** GO analysis for TLPF32h-VS-control. **b** GO analysis for TLPF128h-VS-control. **c** GO analysis for TLPF128h-VS- TLPF32 h

Screening of DEPs was carried out in order to determine which proteins were expressed with a fold change > 1.5 and a Q value < 0.05. We identified 615 DEPs in the TLPF32h group, including 385 up-regulated proteins and 230 down-regulated proteins. There were 907 DEPs in the control group compared with TLPF128h, including 249 up-regulated proteins and 458 down-regulated proteins. As seen in Fig. 6, up-regulation of proteins was more common after the addition of TL, but the number of down-regulated proteins was higher in the TLPF128h group compared to the TLPF32h group.

**Fig. 7:**
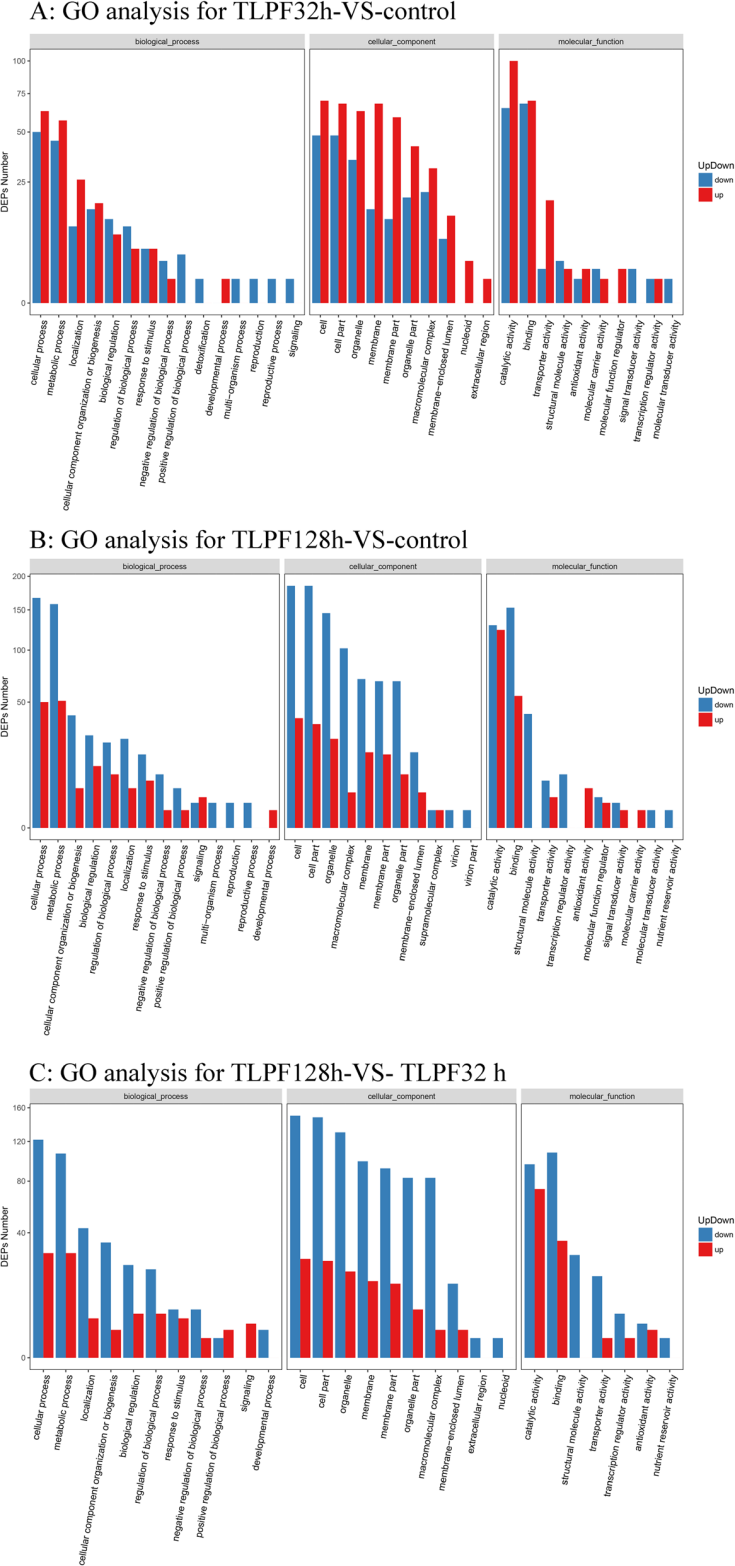
Gene ontology (GO) analysis between groups. A: the KEGG pathway analysis for TLPF32h-VS-control. B: the KEGG pathway analysis for TLPF128h- VS-control. C: the KEGG pathway analysis for TLPF128h-VS- TLPF32 h

Gene ontology (GO) is an important element of bioinformatics research that aims to unify analyses of gene expression and gene attributes of all species. GO annotation analysis showed that 297 DEPs in the TLPF32h group and control group were annotated as belonging to 35 functional groups (Fig. 7). Briefly, the DEPs were found to be mainly involved in cellular processes (38.05%) and metabolic processes (34.34%). Among the cell components, DEPs were mainly concentrated in cells (39.73%) and cell parts (39.06%). In terms of molecular function, DEPs were mainly involved in catalytic activity (55.56%) and binding activity (46.46%).

These results indicated that TL-reactive proteins may be primarily involved in stress responses, chemical stimulation responses, primary metabolic processes, cell responses, and other related functions. We might expect that when *P. igniarius* experiences changing environmental stimuli, the defense system would immediately respond, increasing metabolic activity, producing defensive substances, and enhancing the activity of various enzymes to promote defense.

**Fig. 8:**
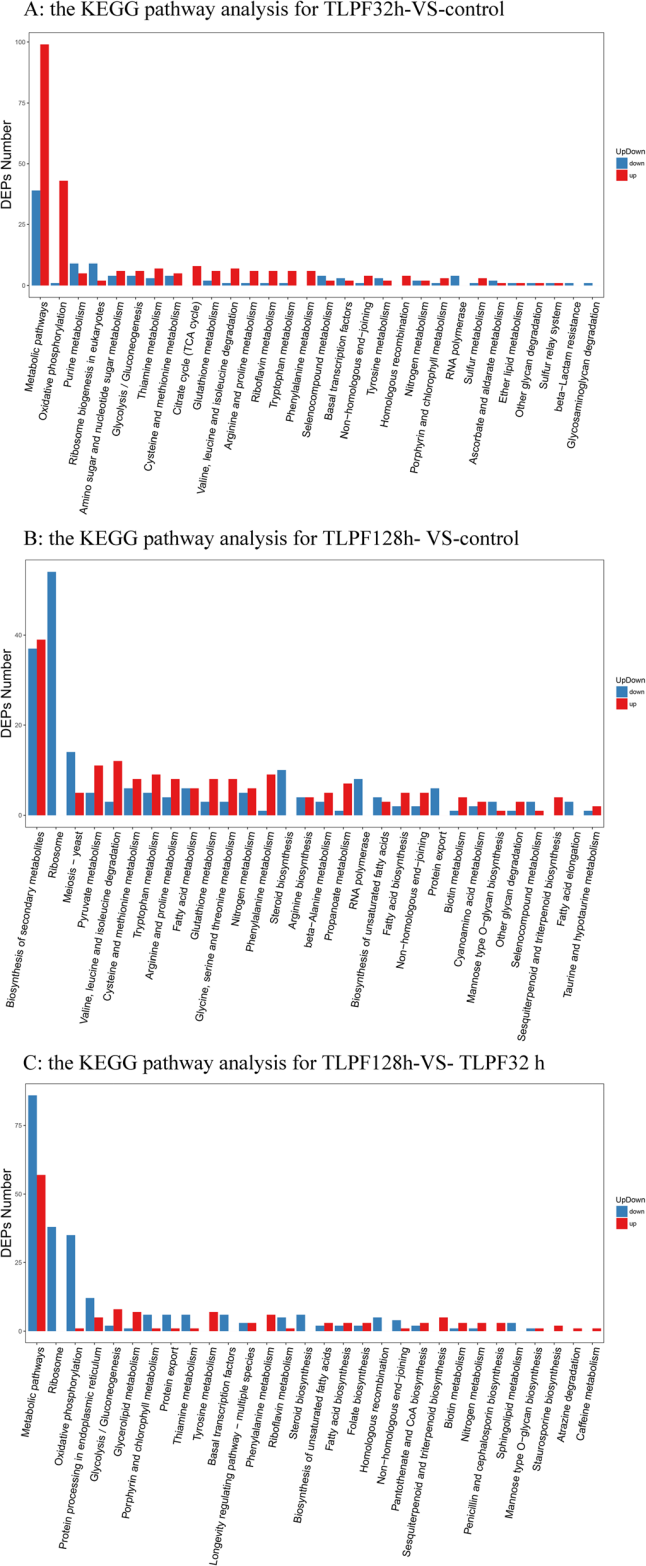
The KEGG pathway analysis between groups

In KEGG pathway enrichment analysis, a total of total 82 DEPs were matched with the KEGG pathway database using Blast_v2.2.26 software. Compared with the control group (Fig. 8), 265 DEPs were labeled as 52 KEGG pathways, and metabolic pathways (ko01100, 77 DEPs) were the primary pathways that were enriched. Indeed, the DEPs were clearly concentrated in metabolic pathways and oxidative phosphorylation, and most of these were up-regulated. Thus, it appears that TL supplementation accelerates *P. igniarius* metabolism and produces oxidative stress. The other DEPs that were identified were mainly related to secondary metabolites and ribosomal protein biosynthesis in the TLPF128h group. Ribosomal proteins represent a common type of RNA-binding protein, which mainly participate in protein translation, post-translational modification, or protein folding. Because we found that the ribosomal protein pathway was down-regulated, we hypothesize that the synthesis of new protein would be slowed. Compared with the TLPF32h group, metabolic processes and ribosomal proteins were the differentially regulated main pathways in the TLPF128h group. Most of the differentially expressed proteins (DEPs) in the 128 h group were down-regulated relative to 32 h, suggesting that the metabolic rate decreased over time.

### Selective verification of iTRAQ data by mass spectrometry multi-reaction monitoring technology (MRM)

In light of the above protein analysis results, 15 candidate DEPs related to the TL reaction were selected to establish an MRM method. Of the 15 target proteins, 11 had MS/MS spectra and unique peptide(s). Therefore, those 11 DEPs were detected and quantified by MRM (Table 2). The results showed that the expression levels of DEP and iTRAQ were almost in direct agreement with each other.

**Table 2:** Quantity analysis of the DEPs

no.	Protein ID	Definition	Fold change			MRM fold change		
			32 h/control	128 h/32 h	128 h/control	32 h/control	128 h/32 h	128 h/control
1	GME472_g	hypothetical protein	2.66	2.25	5.18	7.02	2.25	15.80
2	GME555_g	HSP20 family protein	4.23	–	4.57	23.58	–	19.44
3	GME1626_g	F-type H+ − transporting ATPase	2.79	0.61	1.40	4.22	0.48	2.03
4	GME2462_g	hypothetical protein	3.33	–	3.64	5.36	–	6.64
5	GME3621_g	hypothetical protein	3.07	2.21	6.37	10.18	2.25	22.86
6	GME5465_g	F-type H + - transporting ATPase	3.32	0.64	1.90	3.62	0.36	–
7	GME6086_g	hypothetical protein	2.76	2.52	5.64	6.29	2.33	14.68
8	GME6848_g	GroES-like protein	1.98	1.81	3.50	3.15	2.38	7.50
9	GME8204_g	hypothetical protein	2.25	1.65	3.65	6.91	1.80	12.41
10	GME8292_g	GroES-like protein	2.20	2.82	5.99	5.66	3.51	19.85
11	GME10204_g	hypothetical protein	1.70	1.77	3.08	1.24	3.19	3.94

## Discussion

Genomic mining and functional determination are commonly used to study the mechanisms of fungal biosynthesis and molecular composition. The synthetic genes and regulatory mechanisms of some fungal products have been elucidated in detail, such as monacolin K [24] and griseofulvin. Hispidin and its derivatives are active constituents of edible fungi [25]. Proteomic analysis is an important element of mechanistic studies because of the uncertainties involved with genome sequencing and biosynthetic pathways of compounds. This method was utilized in early studies of the biosynthesis of aflatoxin, and the biosynthesis mechanism was confirmed by feeding the intermediate versicolorin A and using LC-MS/MS to analyze the resulting proteins [26].

In this study, the proteins involved in the biosynthesis of secondary metabolites were identified by iTRAQ method for the first time, and a large number of DEPs were detected in *P. igniarius* after supplementation with the precursor molecule TL. In addition, there were 270 DEPs that were separated into groups including energy metabolism, oxidative phosphorylation, and environmental stress responses. These proteins may provide a new perspective for the study of the biosynthesis of hispidin and its derivatives. Comparative analyses showed that DEPs found after TL feeding were mainly involved in cell processes, metabolic processes, located in cells or cell parts, catalytic activities, and binding functions.

### Comparing the proteomic changes after TL supplementation

After comparing the dynamic changes in concentration of HIP, it appeared that TL supplementation led to significant improvement of HIP production. KEGG pathway enrichment analysis of three groups indicated that DEPs of TLPF32h vs control group were most closely associated with the metabolic pathway (Fig. 8). Almost all proteins had high expression levels that participated in oxidative phosphorylation. The proton pump F-ATPase is an ATP synthase, which plays an important role in energy transduction in most species. Further, the F-ATPase helps maintain the pH of the cytoplasm by secreting protons, and F-ATPase inhibitors reduce the acid resistance of bacteria [27]. In this study, a large number of F-ATPase were up-regulated after TL feeding. The enhancement of ATP synthase, consistent with increased expression of proteins related to oxidative phosphorylation, could increase the ATP supply to the biosynthesis of HIP. Consistently, proteomic analysis showed that NADH dehydrogenase related energy production were also up-regulated in *P. igniarius*. NADH dehydrogenase is the major element of respiratory chain complex I, Electrons mainly move from NADH to the mitochondrial respiratory chain. NADH dehydrogenase is a small protein (< 10 kDa) and relatively simple in structure [28]. Its sub-structures and functional model have been well established. In this study, several complex I subunits were up-regulated to produce more protons, which promote ATP synthesis. That is to say, in *P. igniarius*, increased synthesis of ATP may play a central role in the differential expression seen after supplementation with TL. This ATP-related enzyme was down-regulated on KEGG pathway enrichment analysis of TLPF128h vs the TLPF32h group. The inexistence of riboflavin, thiamine, glutathione, cysteine and methionine, and selenocompound metabolism at TLPF128h vs TLPF32h group showed mycelium metabolism basically returned to normal. It is worth noting that proteins related to ribosomes were significantly down-regulated in TLPF128h. This suggest that synthesis of protein was decreased at that time.

### Environmental stress may lead to the accumulation of HIPs

**Fig. 9:**
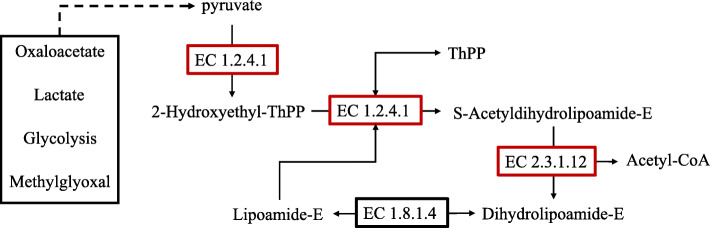
Representative metabolic pathway maps of differentially expressed proteins involved in pyruvate metabolic in KEGG. The enzyme codes were noted as follows: pyruvate dehydrogenase (EC 1.2.4.1), dihydrolipoyllysine-residue acetyltransferase (EC 2.3.2.12), dihydrolipoamide dehydrogenase (EC 1.8.1.4). Red for upregulated proteins

The regulatory mechanisms of macrofungi are largely unclear because of the complexity of the physiological process and genomes. In past years, the development of proteomics has provided insights and a systematic understanding of changes in filamentous fungi and yeast at the molecular level in response to environmental changes, such as the response of *Flammulina velutipes* to cold and light stress [29]. Among the 270 DEPs, some were stress response proteins produced by organisms in response to environmental changes. For example, cysteine proteases respond to biological and abiotic stressors in the external environment. They not only participate in the regulation of transcription factors, but also process proteins [30]. Glutathione S-transferases, which are mainly found in the cytoplasm, are multifunctional supergene family proteins. Fungal glutathione S-transferases have a variety of structures and functions, regulate the growth and development of organisms, and protect the enzyme system from environmental stress [31]. Heat shock proteins (HSPs) were perhaps the most significantly changed DEPs. In emergency circumstances, HSPs help to maintain proper protein folding, promote the transmembrane transport of protein molecules, and promote the recovery or degradation of damaged proteins. In order to promote the hydrolysis of ATP, HSPs activate protein kinase C, thereby reducing the damage caused by oxygen free radicals. Recent research has shown that pyruvate accumulation is a protective mechanism against stress conditions in fungi [32]. In this study, our data showed that pyruvate dehydrogenase and dihydrolipoyllysine-residue acetyltransferase content were increased, probably because of pyruvate accumulation (Fig. 9). These proteins play an important role in the defense response and were up-regulated in TLPF32h. Our previous studies showed that there was trace hispidin and hispidin derivatives in the fermentation broth of *P. igniarius*, found at concentrations too low to be separated and extracted. However, the compounds can be extracted from the fruiting bodies of wild fungi. The dramatic increase in stress proteins indicated that TL feeding caused a stress reaction in *P. igniarius* mycelium. The content of HIP tended to increase over the same timeframe. Therefore, we speculate that the metabolic pathway of hispidin in *P. igniarius* may be related to the accumulation of secondary metabolites in stressful environments.

### Biosynthesis pathway of HIPs

The pathway for biosynthesis of HIP was inferred to be related to the cinnamate pathway for the metabolism of phenylalanine as far back as 1973 [33]. Recent research has revealed that HIP is a precursor of luciferin 3-hydroxyhispidin [34]. It has been found that the luminescence of luminous fungal fruiting body extracts is enhanced by the addition of hispidin biosynthetic components, namely caffeic acid, ATP, and malonyl-CoA [35]. Another theory, that HIP is condensated by TL and 3,4-dihydroxybenzaldehyde based on structural analysis of compounds isolated from the fungus fruiting body [22]. The biogenesis of many HIP derivatives has led to speculation that it is generated by the condensation of HIP, a process that could be catalyzed by peroxidase [36]. The mechanism for synthesis of HIP and other complex derivatives, however, is not specific.

**Fig. 10:**
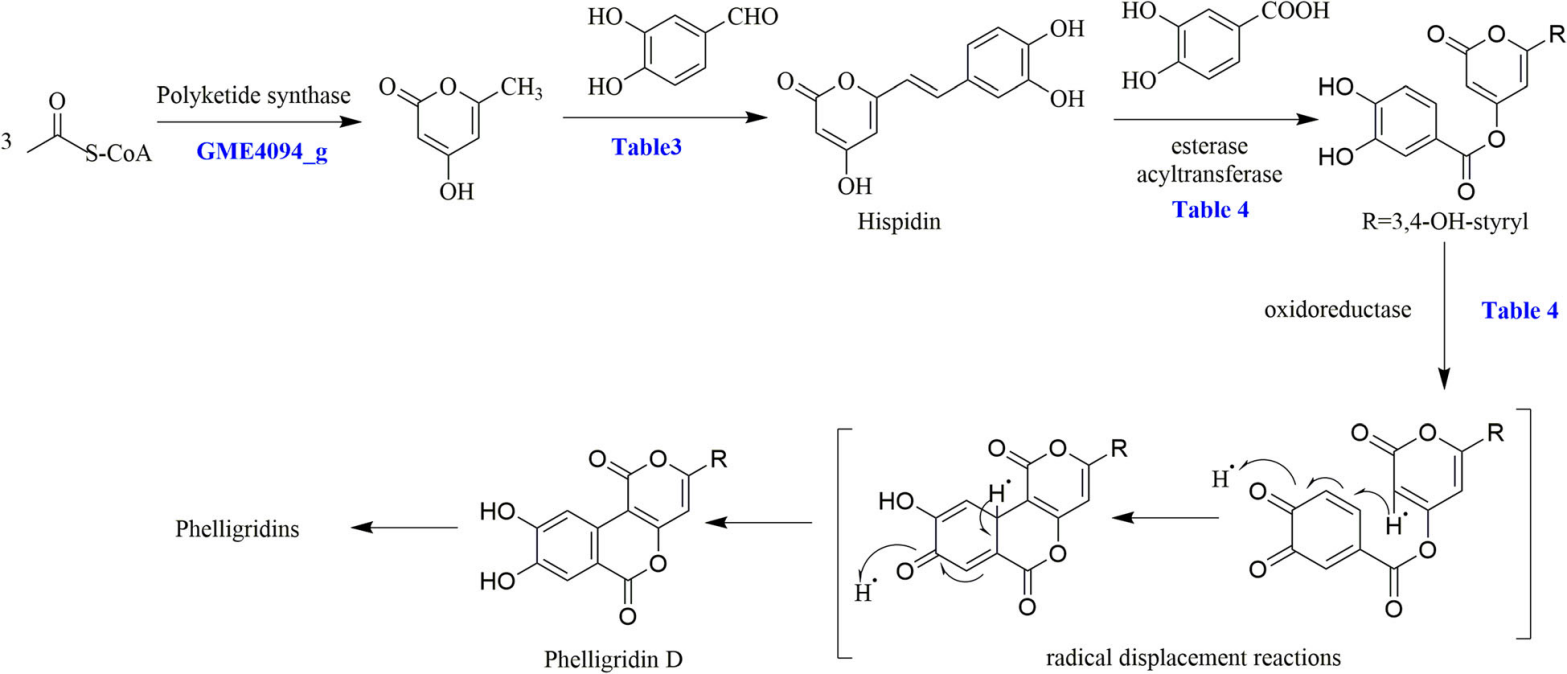
The proposed biosynthesis mechanism of phelligridins

TL is a common, small molecule, polyketone precursor compound that participates in the biosynthesis of HIPs. Expression of the protein GME4094_g, a polyketide synthase, was down-regulated in TLPF32h and not up-regulated in TLPF128h (Table 3). In the synthesis of HIP, dehydrogenase is necessary. Dehydrogenases GME1855_g, GME4242_g, GME4316_g, and GME9582_g were essential in the HIP synthesis pathway and were increased during the HIP production period. A series of enzymes related to the biosynthesis of HIPs is listed in Table 3. In addition, some oxygenases (Table 4) appeared to play a role in the synthesis of HIP’s structural derivatives (such as phelligridin D, Fig. 10). In this process, phenolic compounds are oxidized to benzoquinones that stimulate free radical transfer and then form a variety of compounds with novel chemical skeletons. With further elucidation of the secondary metabolites and regulation of its biosynthetic pathway, it will be increasingly possible to control the production of hispidin via genetic engineering.

**Table 3:** The DEPs related to the biosynthesis of hispidin

no.	Protein ID	Definition	Fold change		
			32 h/control	128 h/32 h	128 h/control
1	GME4094_g	ketoacyl-synt-domain-containing protein	0.64	–	–
2	GME417_g	phenol 2-monooxygenase	–	–	1.52
3	GME418_g	hypothetical protein	2.06	–	2.67
4	GME10220_g	hypothetical protein	1.69	–	2.73
5	GME10204_g	hypothetical protein	1.7	1.77	3.08
6	GME7557_g	3-oxoacyl-[acyl-carrier protein] reductase	1.71	0.64	–
7	GME1172_g	NADH dehydrogenase (ubiquinone)	3.57	–	–
8	GME2933_g	NADH dehydrogenase (ubiquinone)	2.17	0.51	–
9	GME4242_g	Aldo/keto reductase family proteins	1.62	1.83	2.92
10	GME7978_g	2-oxoglutarate dehydrogenase	1.69	–	–
11	GME8093_g	NADH dehydrogenase (ubiquinone)	2.36	–	0.53
12	GME4762_g	NADH dehydrogenase	–	0.6	–
13	GME6232_g	NADH dehydrogenase	2.2	0.48	–
14	GME1172_g	NADH dehydrogenase	3.57	0.57	–
15	GME316_g	NADH dehydrogenase (ubiquinone)	2.31	–	–
16	GME4762_g	NADH dehydrogenase	–	0.6	–
17	GME6848_g	GroES-like protein	1.98	1.81	3.5
18	GME8093_g	NADH dehydrogenase (ubiquinone)	2.36	0.53	–
19	GME4242_g	Aldo/keto reductase family proteins	1.62	1.83	2.92
20	GME2933_g	NADH dehydrogenase (ubiquinone)	2.17	0.51	–

**Table 4:** The DEPs related to the biosynthesis of phelligridins

no.	Protein ID	Definition	Fold change		
			32 h/control	128 h/32 h	128 h/control
1	GME1855_g	ester dehydrase-isomerase	1.65	–	–
2	GME9431_g	acetate-hydrolyzing esterase	–	1.7	2.01
3	GME3566_g	carnitine *O*-acetyltransferase	–	1.53	1.53
4	GME2222_g	gibberellin 2-oxidase	–	1.55	–
5	GME1247_g	NADPH oxidase	–	1.57	–
6	GME4242_g	2-dehydropantolactone reductase	1.62	1.83	2.92
7	GME1010_g	aldo/keto reductase family proteins	–	1.65	–
8	GME4316_g	NADPH_2_ dehydrogenase	–	3.79	4.22
9	GME1973_g	sulfonate dioxygenase	1.64	1.57	–
10	GME202_g	NADH-ubiquinone oxidoreductase	–	1.81	–
11	GME7140_g	predicted NAD-dependent oxidoreductase	3.8	2.59	–
12	GME272_g	norsolorinic acid ketoreductase	–	2.91	3.05
13	GME4319_g	NADH:flavin oxidoreductase	–	1.79	2.69
14	GME4553_g	alkylphenol/PAH-inducible cytochrome P450 monooxygenase	–	1.93	–
15	GME10364_g	norsolorinic acid ketoreductase	–	2.56	2.01
16	GME10320_g	oxidoreductase	2.08	–	2.02
17	GME10204_g	phenol 2-monooxygenase	1.7	–	3.08
18	GME11230_g	NADPH_2_:quinone reductase	1.53	1.62	2.59
19	GME111_g	aldo-keto reductase	–	–	1.68
20	GME6807_g	cytochrome P450 monooxygenase	–	–	1.86
21	GME9582_g	NADPH_2_ dehydrogenase	–	–	1.56
22	GME10875_g	oxidoreductase	1.55	–	1.54
23	GME7140_g	predicted NAD-dependent oxidoreductase	–	2.59	3.8
24	GME9574_g	NADPH_2_ dehydrogenase	–	–	1.59
25	GME10600_g	diacetyl reductase	–	2.02	2.04
26	GME10220_g	phenol 2-monooxygenase	1.69	–	1.69
27	GME7574_g	FAD-dependent monooxygenase	–	–	1.61
28	GME2709_g	NADPH_2_ dehydrogenase	–	–	1.84
29	GME10364_g	norsolorinic acid ketoreductase	–	2.56	2.01
30	GME606_g	NADPH_2_:quinone reductase	–	3.49	2.91
31	GME4315_g	NADPH_2_:quinone reductase	–	1.75	2.17
32	GME1966_g	Aldehyde dehydrogenase	–	–	1.58
33	GME2610_g	FAD-linked oxidoreductase	1.56	–	1.52
34	GME10317_g	monooxygenase	1.74	1.8	3.08

## Conclusion

In this study, a new synthetic pathway of hispidin was analyzed and validated by the precursor feeding method. The DEPs of *P. igniarius* after TL feeding were analyzed using the iTRAQ technique, and DEPs involved in the biosynthesis of hispidin and its derivatives were subsequently screened. The synthesis of hispidin and its derivatives appears to be related to a large number of oxidoreductases and dehydrogenases as well as to stress response systems. According to the results of proteomic analyses and previous data, it appears that TL affects the biosynthesis of hispidin through several routes, including by acting on reactive oxygen species scavenging, signaling pathways, secondary metabolite synthesis, and other reactions. This work sought to provide further mechanistic clarification for the biosynthetic pathways important for the production of hispidin and its derivatives through analysis of whole gene sequences, functional gene annotations, and prediction of secondary metabolite synthesis gene clustering, combined with analysis of DEPs from iTRAQ results. Further research should focus on the biosynthetic gene cluster and synthetase of hispidin.

## Methods

### Materials

*P. igniarius* (CGMCC 5.95) was purchased from the China Microbial Preservation Center (Beijing, China) and was cultured in modified Martin medium (MMM), including 5 g/L peptone, 1 g/L K_2_HPO_3_, 2 g/L yeast extract, 20 g/L glucose, 20 g/L agar, pH 6.2–6.5. The culture medium was sterilized by autoclave at 121 °C for 30 min. The strain was stored at 4 °C.

### HPLC detection of metabolites by precursor feeding

*P. igniarius* was cultured in MMM medium for seven days at 28 °C. An agar patch (1 cm × 1 cm) was added into a 250 mL conical bottle containing 150 mL liquid medium (the above MMM without agar). The culture conditions were 28 °C, 180 rpm. Then, 15 days later, the TL, which was dissolved in double distilled water, was added into the fermentation liquor at a final concentration of ~ 0.1 mg/mL. The co-culture was then allowed to grow for another 9 days under the same conditions. Samples (2.0 mL) were taken at 1, 3, 5, 7, and 9 days after the addition of TL, and were extracted by EtOAc (3 × 2.0 mL). The organic layer was removed by volatilization and the residue was dissolved in 2 mL methanol for HPLC detection. HPLC was conducted using Agilent 1220 Infinity II equipment with Agilent Eclipse XDB-C18 (4.6 mm × 250 mm, 5 μm) as the separation column. Two-phase gradient elution methods were used in this work including 0.2% formic acid (an aqueous solution) as phase A, and MeOH as phase B. The MeOH fraction in phase B was gradually increased from 40% (V/V) to 80% (V/V) over 25 min. The spectra were recorded by a DAD detector at 380 nm. The flow velocity was set to 1 mL/min, and the injection volume was 10 μL.

### Separation and identification of metabolites

A 5000 mL scale-up of *P. igniarius* was cultured according to the above-mentioned fermentation conditions. After the 0.1 mg/mL TL was added, the co-culture was grown for another 3 days. The filtrate of the fermentation broth was concentrated to 500 mL and partitioned with EtOAc (3 × 500 mL). The EtOAc extract was then evaporated under reduced pressure to yield 290 mg of residue, which was subjected to Sephadex LH-20 column chromatography, eluted with petrol-CHCl_3_-MeOH (5:4:1) to produce five fractions (A-E). Fraction B was purified through reverse-phase preparative HPLC using a mobile phase of MeOH-H_2_O (45:55) to afford compound **A** (25.5 mg). Fraction D was separated by preparative RP-HPLC using MeOH-H_2_O (62:38) to afford compound **B** (7.0 mg). The structures of the target compounds were identified by 1D and 2D NMR as well as HRESIMS analysis. 1D- and 2D-NMR spectra were obtained at 400 MHz for ^1^H and 100 MHz for ^13^C, respectively, on Bluker 400 MHz spectrometers in methanol-*d*_4_ with solvent peaks used as references. HRESIMS data were measured using an Agilent 1290 Infinity II Accurate Mass Q-ToF LC/MS spectrometer.

### Determination of HIP yield by external standard method

The concentrations of HIP and TL were determined by HPLC with an external standard at the wavelength of 254 nm, and a 20–80% methanol gradient was used for elution. The concentration points for the HIP standard curve were 1.0, 0.5, 0.25, 0.125, 0.0625, and 0.03125 mg/mL, and the concentrations for the TL standard curve were 8, 4, 2, 1, 0.5, and 0.25 mg/mL. The linear standard equation was obtained by using the linear least square method. As in the previous methods description, *P. igniarius* was cultured in MMM liquid medium for 15 days and then fed TL at a final concentration of 0.1 mg/mL. Fermentation broth samples were taken at 0 h, 1 h, 2 h, 4 h, 8 h, 32 h, 64 h, and 128 h after the addition of TL into the fermentation system, and then the samples were extracted with ethyl acetate and analyzed by HPLC.

### Protein extraction and enzymatic hydrolysis

A 5 mm magnetic bead and 25 μL of lysis buffer were added to 5 mg mycelium samples. The final concentrations of PMSF and EDTA were 1 mM and 2 mM, respectively. The mycelium samples were allowed to rest for 5 min after the eddy oscillation, and the final concentration of DTT was 10 mM. The supernatant was collected by centrifugation at 4 °C for 20 min after 2 min oscillation with a tissue abrasive apparatus. The supernatant was treated with 10 mM DTT for 1 h in a water bath at 56 °C. After coming back down to room temperature, IAM was added at a final concentration of 55 mM, and then the sample was kept in the dark for 45 min. The supernatant was precooled with 5× volume of acetone, precipitated at − 20 °C for 2 h, and was centrifuged at 9000×g, 4 °C for 20 min. The addition of acetone was repeated three times, followed each time by centrifugation and discarding the supernatant until the supernatant was colorless. Then, 25 μL of lysis buffer was added into the precipitation mixture. After 5 min of ultrasonication in an ice bath, the supernatant was centrifuged at 9000×g, 4 °C for 20 min.

### iTRAQ marker and peptide segment separation

First, 100 μg protein solution was extracted from each sample, and the trypsin enzyme was added in the ratio of protein:enzyme of 40:1. The enzyme was hydrolyzed for 4 h at 37 °C, at which point trypsin was again added (the same amount), and the mixture was continuously hydrolyzed for another 8 h. The resulting enzymatically generated peptides were desalted using a Strata X column and then vacuum-dried.

Eight groups of iTRAQ labeling reagents (113, 114, 115, 116, 117, 118, 119, 121) were selected. Then, 50 μL isopropanol were added to each tube at 25 °C, and the mixture was centrifuged after swirl oscillation. The supernatants were transferred to another clean sample tube along with the enzymatic peptides in 0.5 M TEAB. The different peptide fragments were labeled with their respective peptide’s labels.

The above samples were separated by liquid phase chromatography with a LC-20AB system (Shimadzu, Japan), and the separation column used was a Gemini C18 column (5 μm, 4.6 × 250 mm). The above dried samples were re-dissolved with phase A (5% ACN, pH 9.8), and the flow rate gradient was set as: 5% mobile phase B (95% ACN, pH 9.8) for 10 min, 5–35% mobile phase B for 40 min, 35–95% mobile phase B for 3 min, and 5% mobile phase B for 10 min, at rate of 1.0 × 10^− 3^ L/min. The detection wavelength used was 214 nm. The fractions were collected once per minute, and the sample components were merged by chromatographic elution peaks to yield a total of 20 fractions. Those 20 fractions were frozen and concentrated each to the same volume of 1.0 mL.

### LC-MS/MS analysis

After centrifugation for 10 min, the supernatant samples were separated using LC-20 AD nanoflow liquid chromatography. A trap column was used to concentrate protein and remove salts. It was connected with a self-assembled C18 column (75 μm inner diameter, 3.6 μm column diameter, 15 cm column length) in series. The flow rate was set to 300 nL/min. Separation was carried out using the following gradients:

(1) 0–8 min, 5% mobile phase B (98% acetonitrile aqueous solution containing 0.1% formic acid (FA));

(2) 8–43 min, 8–35% mobile phase B gradient;

(3) 43–48 min, 35–60% mobile phase B gradient;

(4) 48–50 min, 60–80% mobile phase B gradient;

(5) 50–55 min, equivalent 80% mobile phase B;

(6) 55–65 min, equivalent 5% mobile phase B.

After ionization from a nano ESI source, the peptide segments were analyzed by high resolution liquid chromatography-mass spectrometry (HR-LC-MS) TripleTOF 5600. Using Proteome Discoverer, a Thermo Scientific tool, the original mass spectrometry file was converted into a MGF format file containing the information of secondary mass spectrometry (MS/MS) spectra, in which “BEGIN IONS” and “END IONS” were the starting and ending positions of each spectrum. The UniProt protein database was used to identify the proteins. The MGF file and protein database were searched to obtain the final protein identification results using the Mascot 2.3.02 identification software.

### iTRAQ data analysis

iTRAQ data were quantified by IQuant software. The spectra and the list of peptide segments were filtered by 1% FDR (PSM-level FDR < 0.01) to identify significant results. According to the parsimony principle, the peptide segments were used to assemble proteins and produce a series of proteomes. In order to control the false positive rate of proteins, the process was again filtered at the protein level with 1% FDR (Protein-level FDR < 0.01). Quant’s workflow included the following steps: protein filtering, purity correction of report group labels, normalization of quantitative values, complementation of missing values, calculation of quantitative values of proteins, statistical analysis, and display of final results.

### Bioinformatic analysis

In this study, three sets of experiments including Control, TLPF32h, TLPF128h, and three sets of comparisons of TLPF32h /control, TLPF128h/control, TLPF128h/TLPF32h were conducted. The differentially expressed proteins (DEPs) were screened on the basis of a fold change > 1.5 and *P* < 0.05.

In the GO enrichment analysis of DEPs, the GO entries with significant enrichment were identified by hypergeometric test, compared with all identified proteins as background. The principle of Pathway enrichment analysis was similar. The hypergeometric test formula was as follows:

$$ \mathrm{P}=1\hbox{-} \sum \limits_{\mathrm{i}=0}^{\mathrm{m}-1}\frac{\left({}_{\mathrm{i}}^{\mathrm{M}}\right)\left({}_{n-i}^{N-M}\right)}{\left({}_n^N\right)} $$

N: The number of GO entries matched in all identified proteins; n: the number of GO entries matched in DEPs; M: one of the GO entries matched in all identified proteins; m: one of the GO entries matched in DEPs. DEPs were determined to be significantly enriched in the GO entry if the *P* value of the hypergeometric test was less than 0.05.

### Validation of MRM technology

According to the results of iTRAQ data, differentially expressed proteins were screened and selected for MRM validation. Each sample was treated with 100 μg protein solution. Trypsin enzyme was added at a ratio of protein:enzyme of 40:1, with a total amount of 2.5 μg of trypsin enzyme, and enzymatic hydrolysis was carried out at 37 °C for 4 h. Trypsin was added one more time at the same ratio, and the enzymatic hydrolysis was continued at 37 °C for another 8 h. The enzymatic peptides were desalted using a Strata X column and then vacuum-dried.

The samples were scanned with a HPLC-TripleTOF 5600 mass spectrometer, and the resulting data were searched by Mascot v2.3 using the Fungi protein database (11,243 sequences) added to the internal standard peptide sequence. The DAT files were imported into Skyline software to establish the atlas library (credibility > 0.95). Skyline software was used to select target peptide segments under the following conditions:

(1) The peptide segments had matched second-order ions.

(2) The length of the peptide segments were between 5 and 40 amino acids.

(3) The peptide segment was the only one for the target protein.

(4) The cysteine in the peptide segment was modified to carbamidomethyl.

(5) There was no variable modification of the peptide segment.

(6) No methionine was found in the peptide segment.

(7) There was no missing cut of the peptide segment.

Transition selection set was determined as follows:

(1) The fragment ions were B and Y ions;

(2) The parent ion charges were 2,3,4;

(3) The charges of fragment ions were 1,2;

(4) Debris ion charge-mass ratio < 1250 (four-stage rod scanning range);

(5) The number of transitions was 6.

### Validation of MRM method

Skyline set up the MRM method and output it to the QTRAP 5500 mass spectrometer for MRM scanning verification. The success of MRM mass spectrometry for target proteins depended on the validation results, which must conform to:

(1) There were co-elution peaks in transitions with different peptide segments.

(2) The chromatographic peak area intensity of transitions with different peptide segments was correlated with the fragmentation intensity of spectral data.

(3) The retention time of the peptide segments in MR and full-spectrum scans was good.

### MRM mass spectrometry detection

The peptide fragments were separated by liquid phase and entered into the QTRAP 5500 tandem mass spectrometer. The ion source was Nanospray Illsource. In data acquisition, the instrument parameters are set as follows: spray voltage was 2400 V, spray gas was 23. With MRM scanning mode, the resolution of Q1 and Q3 was set to Unit mode.

### Data analysis

Each transition signal of the target protein was normalized to the signal of beta-galactosidase. After normalized intensity, a linear mixed model integrated with the MS stats tool was used to quantify the target protein in the sample. This model gave the ratio of protein in the comparison group and the adjusted *p*-value. The corrected *P* value reflected the false positive rate of the original statistical test (Benjamin and Hochberg). If the difference in final target protein concentration was at least 1.5-fold with *P* < 0.05 (false positive < 0.05), the protein was considered to be significantly differentially expressed.

## Supplementary Information

**Additional file 1: Figs. S1–9.** MS, 1D and 2D NMR spectra of compounds **A** and **B**. This material is available free of charge via the Internet at http://www.sciencedirect.com.

## Data Availability

The datasets used and/or analysed during the current study are available from the corresponding author on reasonable request.

## Abbreviations

ACN: Acetonitrile
ATP: Adenosine triphosphate
DAD: Diode array detector
DEPs: Difference expression of proteins
DTT: Dithiothreitol
EDTA: Ethylene diamine tetraacetic acid
FDR: False discovery rate
GO: Gene ontology
HIP: Hispidin
HMBC: Heteronuclear multiple bond correlation
HPLC: High performance liquid chromatography
HRESIMS: High resolution electrospray ionization mass spectroscopy
HSP: Heat shock protein
HSQC: Heteronuclear single quantum correlation
IAM: Iodoacetamide
KEGG: Kyoto encyclopedia of genes and genomes
MMM: Modified Martin medium
MRM: Multiple reaction monitoring
NMR: Nuclear Magnetic Resonance
PMSF: Phenylmethanesulfonyl fluoride
TEAB: Tetraethylammonium bromide
TLPF: Tricetolatone precursor feeding
