# Supplementary material for: Screening for proteins related to the biosynthesis of hispidin and its derivatives in Phellinus igniarius using iTRAQ proteomic analysis
Source: BMC Microbiol. 2021 Mar 12;21:81. doi: 10.1186/s12866-021-02134-0 (PMC7953727; doi:10.1186/s12866-021-02134-0)
Supplement: Supplementary file 1 — Additional file 1: Figs. S1–9. MS, 1D and 2D NMR spectra of compounds A and B. This material is available free of charge via the Internet at http://www.sciencedirect.com. [file 12866_2021_2134_MOESM1_ESM.doc]

**Screening for hispidin biosynthetic process related protein via iTRAQ proteomic analysis in *Phellinus igniarius***

**Jinjing Guo^a^, Xiaoxi Liu^a#^, Yuanjie Li^a#^, Hongyan Ji^b^, Cheng Liu^a^, Li Zhou^a^, Yu Huang^a^, Changcai Bai^a^, Zhibo Jiang^c^, Xiuli Wu^a*^**

^a^ *College of Pharmacy, Ningxia Medical University, Key Laboratory of Traditional Chinese Medicine Modernization, Ministry of Education at Ningxia Medical University, Yinchuan, 750004, P.R. China.*

^b^ *Department of Pharmaceutics, General Hospital of Ningxia Medical University, Yinchuan, 750004, P.R. China.*

^c^ *School of Chemistry and Chemical Engineering, North Minzu University; Key Laboratory for Chemical Engineering and Technology, State Ethnic Affairs Commission, North Minzu University, Yinchuan, 750021, P.R. China.*

^#^ These authors contributed equally to this work

***** Correspondence author e-mail: myjustmy@163.com (Xiuli Wu)

All the following figures are from our research results.

List of Contents

| no. | Content | Page |
| --- | --- | --- |
| 1 | Figure S1. The Structure of compound **A** and compound **B** | 2 |
| 2 | Figure S2. The (-)-HRESIMS Spectroscopic Data of compound **A** | 3 |
| 3 | Figure S3. The ^1^H-NMR Spectrum of compound **A** in methanol-*d*_4_ (400 MHz) | 4 |
| 4 | Figure S4. The ^13^C-NMR Spectrum of compound **A** in methanol-*d*_4_ (100 MHz) | 5 |
| 5 | Figure S5. The ^1^H-^1^H gCOSY Spectrum of compound **A** in methanol-*d*_4_ (400 MHz) | 6 |
| 6 | Figure S6. The gHSQC Spectrum of compound **A** in methanol-*d*_4_ (400 MHz for ^1^H) | 7 |
| 7 | Figure S7. The HMBC Spectrum of compound **A** in methanol-*d*_4_ (400 MHz for ^1^H) | 8 |
| 8 | Figure S8. The (-)-HRESIMS Spectroscopic Data of compound **B** | 9 |
| 9 | Figure S9. The ^1^H-NMR Spectrum of compound **B** in DMSO (400 MHz) | 10 |


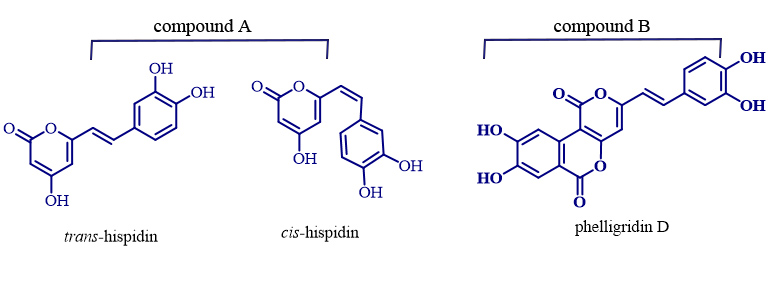


Figure S1. The Structure of compounds **A** and **B**


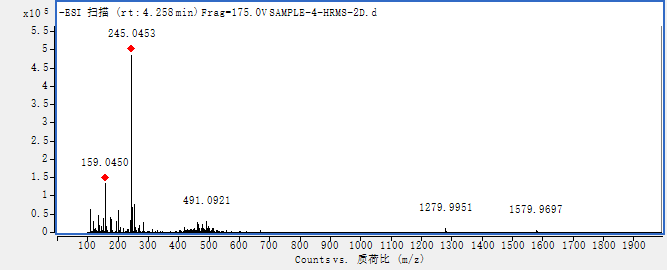


Figure S2. The (-)-HRESIMS Spectroscopic Data of compound **A**


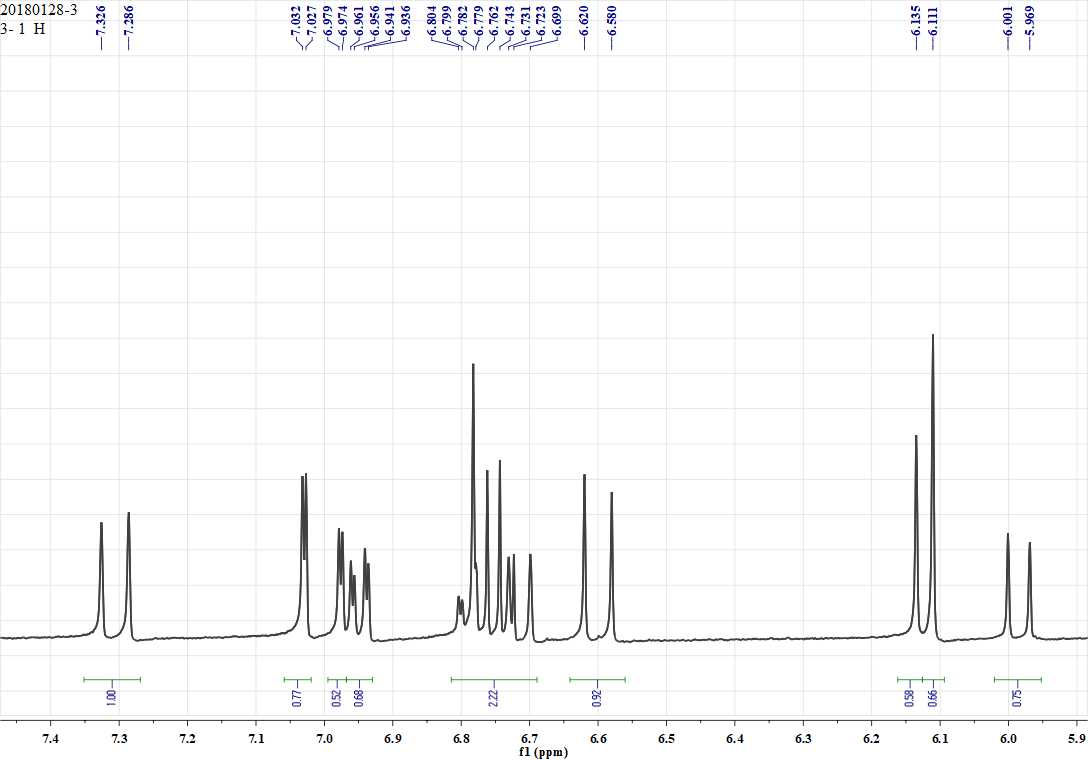


Figure S3. The ^1^H-NMR Spectrum of compound **A** in methanol-*d*_4_ (400 MHz)


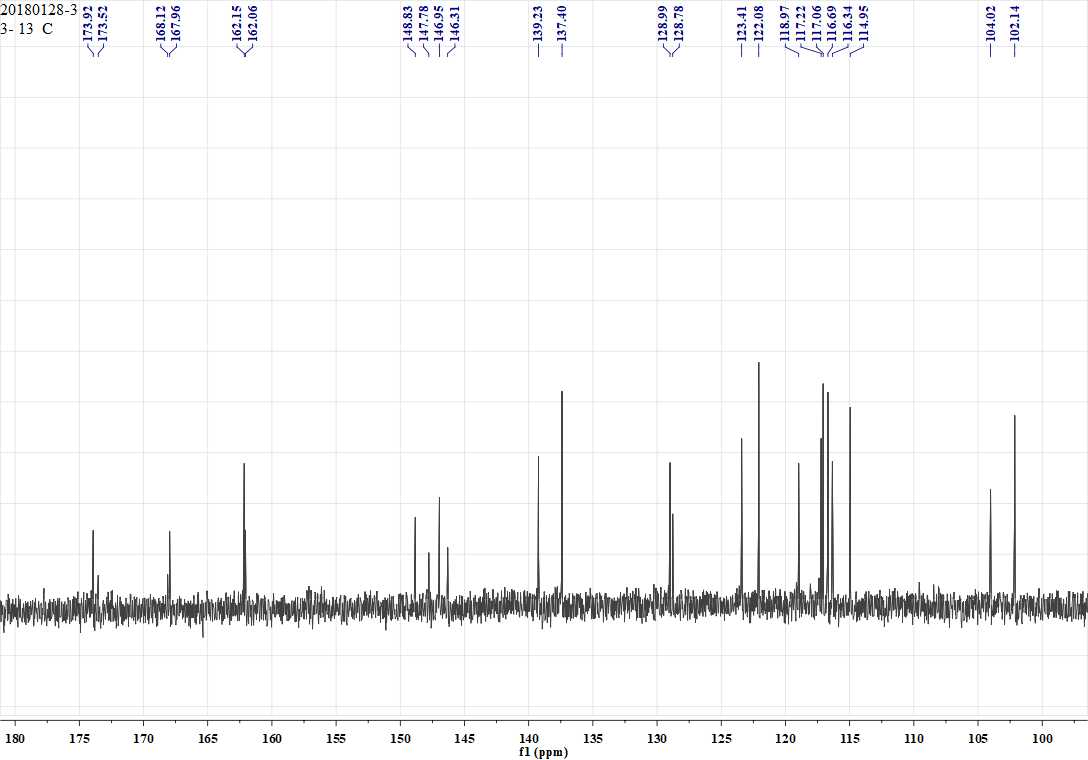


Figure S4. The ^13^C-NMR Spectrum of compound **A** in methanol-*d*_4_ (100 MHz)


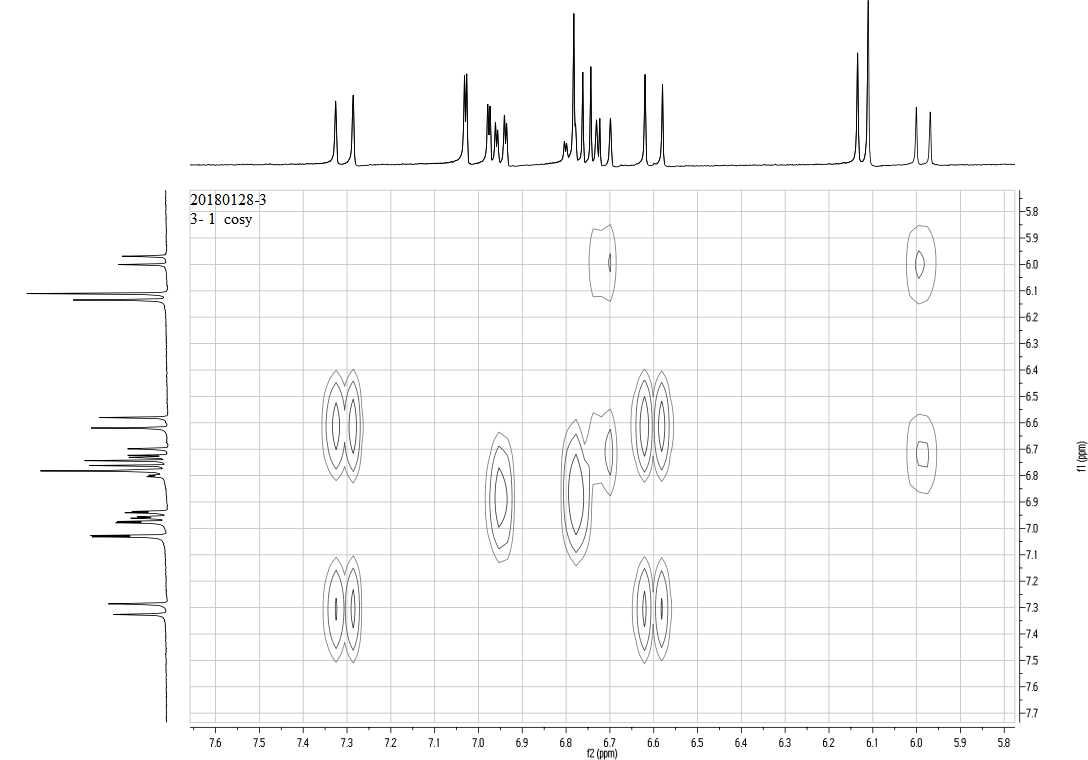


Figure S5. The ^1^H-^1^H gCOSY Spectrum of compound **A** in methanol-*d*_4_ (400 MHz)


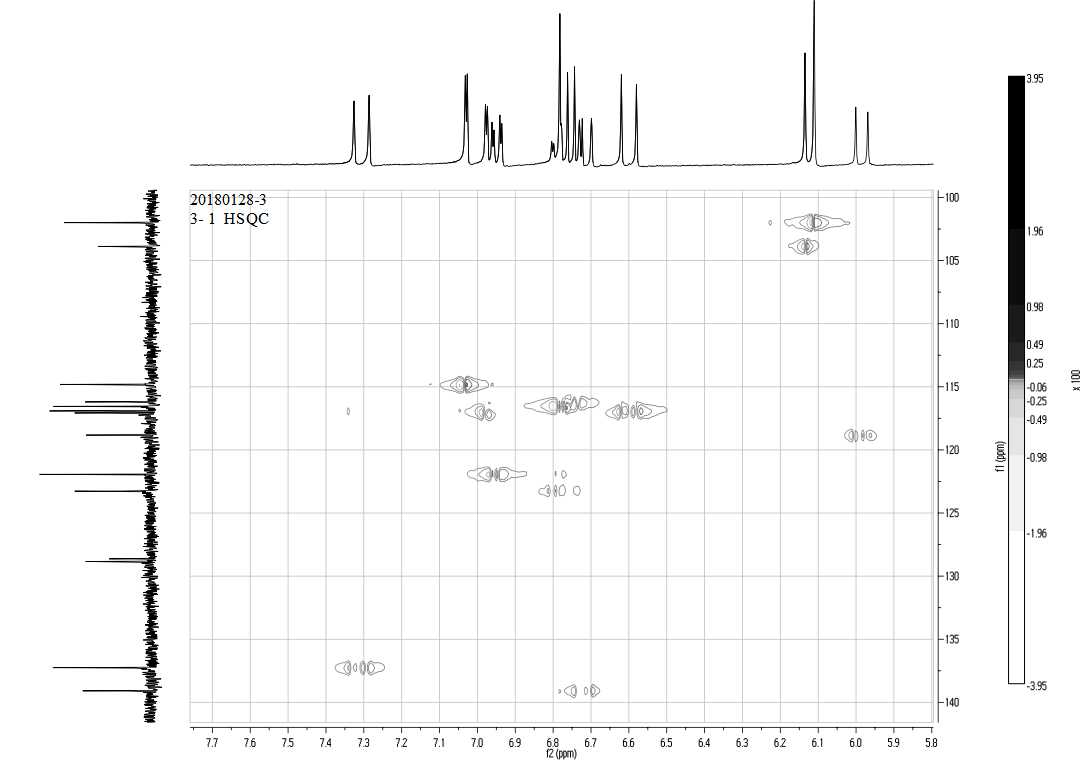


Figure S6. The gHSQC Spectrum of compound **A** in methanol-*d*_4_ (400 MHz for ^1^H)


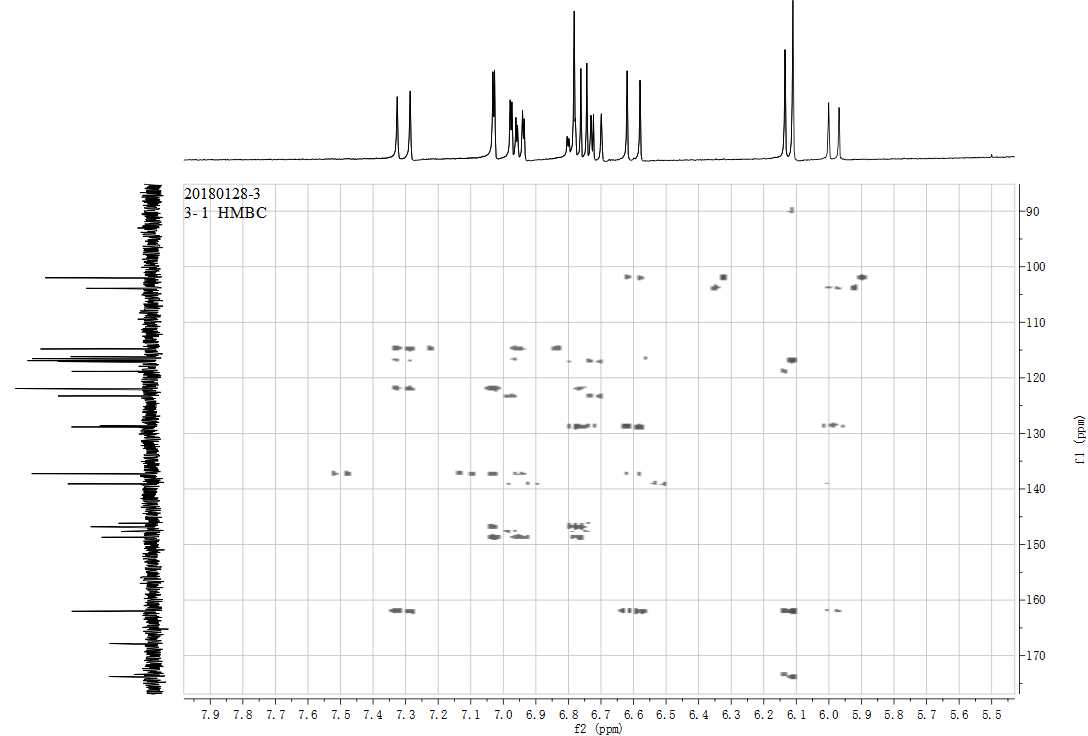


Figure S7. The HMBC Spectrum of compound **A** in methanol-*d*_4_ (400 MHz for ^1^H)


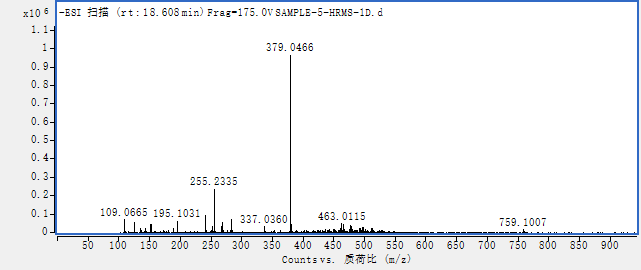


Figure S8. The (-)-HRESIMS Spectroscopic Data of compound **B**


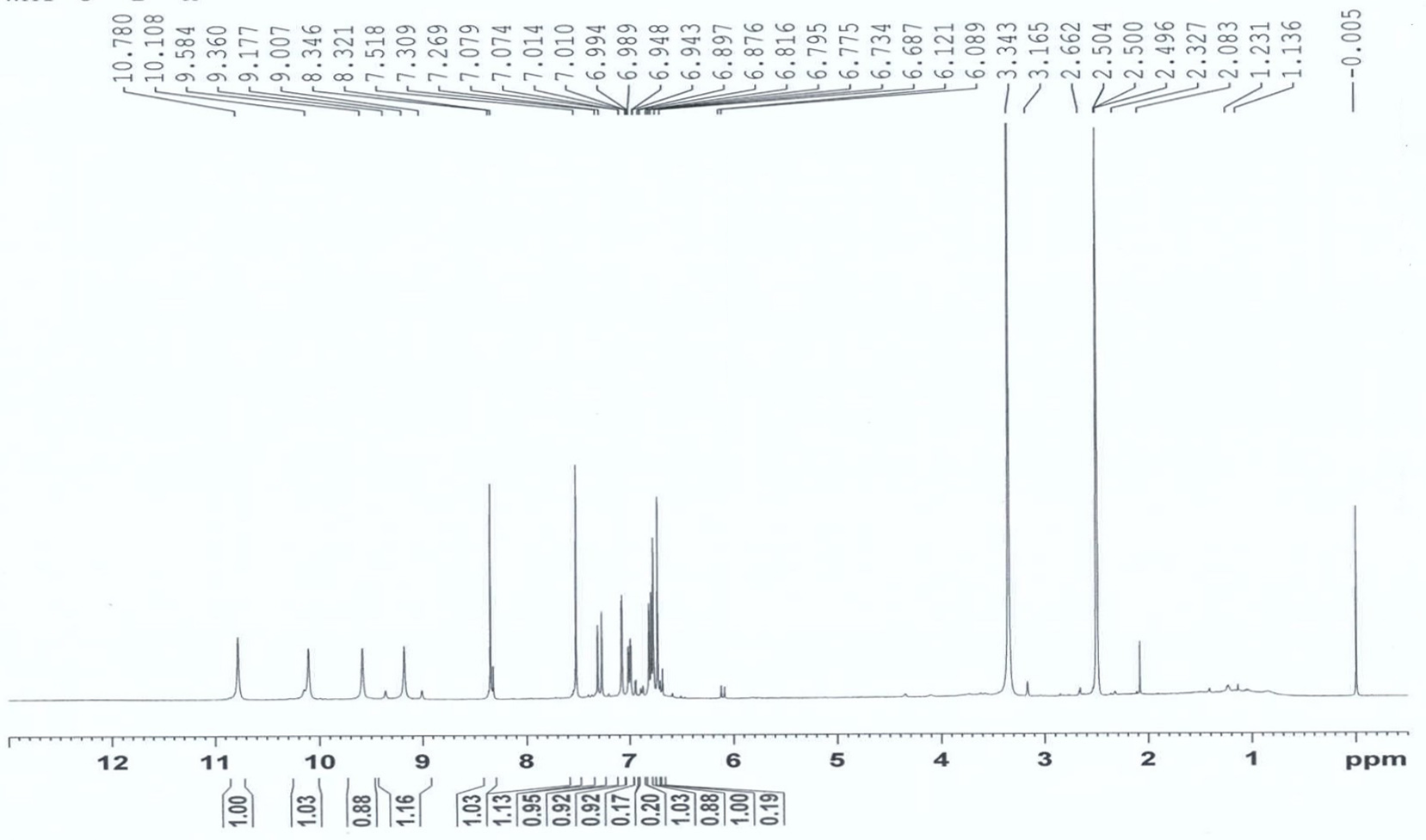


Figure S9. The ^1^H-NMR Spectrum of compound **B** in DMSO (400 MHz)
